# Paradoxical home temperatures during cold weather: a proof-of-concept study

**DOI:** 10.1007/s00484-020-01998-7

**Published:** 2020-08-27

**Authors:** Niilo R. I. Ryti, Anton Korpelainen, Olli Seppänen, Jouni J. K. Jaakkola

**Affiliations:** 1 Center for Environmental and Respiratory Health Research (CERH), Faculty of Medicine, University of Oulu, PO Box 5000, FI-90014 Oulu, Finland; 2 Medical Research Center Oulu, Oulu University Hospital and University of Oulu, Oulu, Finland; 3 FINVAC, Helsinki, Finland

**Keywords:** Health, Heating, Indoor temperature, Outdoor temperature, Temperature, Weather

## Abstract

There is substantial epidemiological evidence on the associations between cold weather and adverse health effects. Meteorological alarm systems are being developed globally, and generalized protective advice is given to the public based on outdoor exposure parameters. It is not clear how these shared outdoor exposure parameters relate to the individual-level thermal exposure indoors, where the majority of time is spent. We hypothesized a priori that there are opposite correlations between indoor and outdoor temperatures in residential apartments. Apartments were classified into 3 categories according to their response to declining outdoor temperature: under-controlled apartments cool down, controlled apartments maintain constant indoor temperature level, and over-controlled apartments warm up. Outdoor and indoor temperatures were measured in 30-min intervals in 417 residential apartments in 14 buildings in Kotka, Finland, between February and April 2018 with outdoor temperatures ranging from − 20.4 °C to + 14.0 °C. Different apartment types were present in all buildings. Floor and orientation did not explain the divergence. Indoor temperatures below the limit value + 20 °C by building code occurred in 26.2%, 7.9%, and 23.6% of the under-controlled, controlled, and over-controlled apartments, some in conjunction with increasing outdoor temperatures. Indoor temperatures above the limit + 25 °C occurred but were more rare. This proof-of-concept study demonstrates that while the home environment may be a source of thermal stress during cold weather, generalized advice for adjusting the heating may lead to paradoxical exposures in some cases. More elaborate conceptualizations of everyday thermal exposures are needed to safely reduce weather-related health risks using shared meteorological alarm systems.

**Electronic supplementary material:**

The online version of this article (10.1007/s00484-020-01998-7) contains supplementary material, which is available to authorized users.

## Introduction

There is substantial global evidence on the associations between outdoor temperature and adverse health effects (Bhaskaran et al. 2009; Guo et al. 2012; Monteiro et al. 2012; Ye et al. 2012; Gasparrini et al. 2015; Ryti et al. 2016; Ryti et al. 2017; Vicedo-Cabrera et al. 2018). Shared outdoor temperatures are commonly used as the predictors of health effects in a population, and the actual individual exposures or places of exposures during the weather events are not known (Ryti et al. 2016).

Major national and international monitoring and public health programs have been developed to mitigate the health effects of harmful weather (Fritsch et al. 2008; Wolf et al. 2010; Conlon et al. 2011; Laaidi et al. 2013; Toloo et al. 2013; Katiyo et al. 2018). For instance, the Network of European Meteorological Services EUMETNET provides real-time warnings of the occurrence, intensity, and substance of extreme weather events occurring throughout Europe (http://www.meteoalarm.eu). The rationale and premise of an alarm system are that an alarm should be followed by effective protective action. The meteorological alarm systems, too, are based on shared exposure parameters such as outdoor temperature. This is necessary since the exposures of the individuals are not known. Epidemiologically thinking, for such a shared system to predict individual exposures and individual health effects so that they could be avoided, the direct health effects of ambient temperature should be induced during time spent outdoors (where the system measures the temperature), or alternatively, there should be a correlation between the outdoor temperature and harmful thermal exposures in other environments such as the home microenvironment. Given the fact that on average over 90% of time is spent indoors (Klepeis et al. 2001; Brasche and Bischof 2005; Matz et al. 2015; Mäkinen et al. 2006), and considering the substantial evidence on the relevance of indoor environment to health (WHO 1987), it seems an oversimplification to consider the shared outdoor environment as only place of relevant thermal exposure during cold weather events.

There is evidence on the association between indoor temperature and adverse health effects (The Eurowinter Group 1997; Marmot Review Team 2011; Jevons et al. 2016). Low living room temperature and inadequate heating of the bedroom are associated with higher winter mortality rate (The Eurowinter Group 1997; Wilkinson et al. 2001), higher blood pressure (Saeki et al. 2014; Shiue and Shiue 2014), health status of COPD patients (Osman et al. 2008), and lung function of asthmatic children (Pierse et al. 2013), and there are descriptions of poor health and a variety of social and economic problems for the residents (Marmot Review Team 2011). Building construction year, poor energy efficiency rating, and fuel poverty have also been associated with excess winter mortality (Wilkinson et al. 2001; Marmot Review Team 2011). The potential effects of high indoor temperature during the heating season have received less attention (Conlon et al. 2011; Jevons et al. 2016). In a series of studies conducted in Finland, room temperature during the heating season was found to be the most important indoor air determinant of sick building syndrome symptoms (Jaakkola et al. 1989; Jaakkola et al. 1991a), i.e., a combination score of various health symptoms of individuals, including nasal, eye, and mucous membrane symptoms; lethargy; skin symptoms; and headache. There was a linear correlation between room temperature above optimal and the score, and individuals exhibited more symptoms at higher room temperatures during the heating season (Jaakkola et al. 1991b)..

Although safe maximum room temperatures during the heating season have been defined in engineering sciences and legislation for decades, they have rarely been implemented in public health programs or weather action plans per se. The latest 2018 Cold Weather Plan for England includes a standardized advice issued by the Met Office and Public Health England during severe cold weather. The advice states, among other things: “heating your home to at least + 18 °C in winter poses minimal risk to your health when you are wearing suitable clothing” (Katiyo et al. 2018). There is no threshold for safe maximum room temperature. The reason for focusing on lower safety thresholds may be that in many countries the building stock and engineering solutions promote positive correlations between outdoor and indoor temperatures. Nevertheless, even in these infrastructures, there are exceptions in the building stock and temperature control systems, and human behavior may also influence the environmental indoor conditions (Keatinge 1986).

It is relevant to ask whether it is safe to provide uniform behavioral advice to all members of the general public, if different individuals may have different or even opposite exposure profiles during the weather events. This could be the case if, for example, some apartments would automatically overheat as response to the cold weather, but everyone would be advised to increase the heating.

We conducted a proof-of-concept study with focus on demonstrating conceptual differences in how apartments react to changes in cold weather. Our general objective is to bridge the gap between the shared weather parameters, which are commonly used as predictors of population-level health effects (Toloo et al. 2013; Vicedo-Cabrera et al. 2018), and the individual thermal exposures at homes, where most of the time is spent (Klepeis et al. 2001). The rationale is twofold. First, preparedness systems rely on shared measurements of shared exposures, and it would be useful to know how these measurements predict the actual conditions in different microenvironments. Second, before providing general advice on safe behavior during weather events, it would be wise to ascertain that following the advice would not lead to additional harms for some individuals.

We hypothesized a priori that there are opposite correlations between indoor and outdoor temperatures in Finnish residential apartments, i.e., while some apartments may cool during cold weather, some may overheat. We classified the apartments based on their responsiveness to weather and assessed how apartments in the different classes performed in terms of providing a healthy thermal environment for the inhabitants.

## Materials and methods

This longitudinal study of residential apartments was conducted in the city of Kotka, Finland, with heating design temperature of − 26 °C, over the time period from February 1 to April 10, 2018.

### Home selection

A total of 417 residential apartments in 14 apartment buildings were included in the study. All buildings were managed by the same property management company, were clients of the same energy company, and were clients of the same Finnish startup company Residentia Ltd., which offers to improve the living comfort and energy expenditure by improved automatic temperature control of the home environment. The measurements for this study were conducted before starting the improved automatic control system; i.e., the apartments were in their normal pre-service state they had been in for years. The apartments did not become clients of these companies because they performed in a particular manner in terms of indoor environmental conditions.

Heat distribution in all apartments was similar and based on hot water circulation, with radiators as the only heat source. There was no floor heating. The temperature control system in all buildings was based on the control of supply water temperature to the radiators and thermostatic radiator valves. Set point of the supply water temperature depended on the outdoor temperature. The water circulation systems have been balanced for each room based on room temperature during the construction phase, whereas the residents can only control the thermostatic radiator valves.

### Data collection

Each apartment was installed a Bosch BME280 environmental sensor (operation range from − 40 °C to + 85 °C, refresh rate 1 Hz, accuracy tolerance ± 3%) to record the indoor air temperature and relative humidity in 30-min intervals. In Finnish buildings, thermal insulation of the building envelope is so good (triple pane windows, maximum of *U* value 1.4 W/m2K of windows and 0.25 W/m2K of walls) that the effect of surface temperature is negligible on operative temperature. This is indicated also by the fact that Finnish building code does not use operative temperature but simply room temperature (Finnish Ministry of Social Affairs and Health 2015). Following a protocol, the sensors were placed 1.6 m above the floor; away from windows and doors leading outside; away from direct sunlight; away from heaters and other sources of thermal energy; away from draft; away from moisture sources from the kitchen, bathroom, and shower; and not in a direct physical contact with the outer wall. The most common placement was the conjunction of the largest open space such as the conjunction of the living room and a hallway.

Each apartment building was installed a Bosch BME280 environmental sensor to record the outdoor temperature and relative humidity in 2-min intervals. Following a protocol, the sensors were placed in the immediate outdoor environment of the building; on the northern wall; away from direct sunlight; and not directly above doors or windows or other openings leading inside the building, i.e., they were free from warm and/or humid air flows originating from indoors. While the shaft of the device was attached to the wall of the building, the actual sensor was not in a direct contact with the wall.

We tried to ensure that recording would have minimal impact on the lives and behaviors of the residents. The recording devices were physically small and minimally visible, and the data gathering process was fully automated. JSON data from the sensors was relayed via Bluetooth to a Raspberry-Pi, which relayed the data to a cloud-based server via SigFox unaltered. We transferred JSON to .csv in R and confirmed data consistency manually and by statistical assessments.

Data on building characteristics such as the construction year, number of apartments, floor area, heating type, maintenance of the heating system, and energy consumption were gathered from records kept by the property management company. Data on apartment floor, orientation, and heating degree days (HDD) was provided by Residentia Ltd. HDD describes the heating need of buildings over a period of time, usually 1 year. It is the sum of temperature differences between assumed indoor temperatures and real outdoor temperatures.

### Data processing

We linked each time-series of apartment-specific indoor temperatures with the time-series of outdoor temperatures of the same building. The direction of hypothesized causal effect was taken into account in the linking process, i.e., outdoor temperature precedes indoor temperature if they were measured at slightly different times. Time difference between these two time-series is ≤ 2 min in our data.

All time-series were checked for outliers and irregularities manually and by statistical assessments. Outliers were found on the outdoor time-series of 2 apartment buildings. A consultation with the startup company confirmed that the respective sensors had been misplaced and exposed to direct sunlight during the daylight hours. We replaced the invalid outdoor time-series with those of the adjacent (closest) buildings. We validated the use of the adjacent time-series by (a) verifying a high correlation between the invalid outdoor time-series and the new outdoor time-series during non-daylight hours and (b) verifying a high correlation between the outdoor time-series of all apartment buildings during all hours.

### Definition of apartment types

Three apartment categories (types) were conceived a priori to address the objectives of the study. The statistical operationalization consisted of two components: (a) direction of cross-correlation coefficient (CCC) between apartment-specific outdoor and indoor temperatures, assessed by cross-correlation functions, and (b) variation of indoor temperatures in the apartment, using 2 °C temperature range as a cut point between thermal stability and lability.

In conjunction with decreasing outdoor temperature, *under-controlled apartments* (type 1) were those with decreasing indoor temperature (positive CCC) and temperature range > 2 °C; *over-controlled apartments* (type 3) were those with rising indoor temperature (negative CCC) and temperature range > 2 °C; and *controlled apartments* (type 2) maintained indoor temperature within the narrow range of 2 °C regardless of the changes in outdoor temperature (direction of CCC not relevant). Strength of CCC was not part of the classification criteria. To exclude influence of outliers in the data, the 2 °C range was assessed by comparing the temperature range and standard deviation (SD) of indoor temperatures in each apartment and selecting the smaller of the two values. Time to maximum correlation (TMC) was allowed to vary between apartments, and the strongest CCC was used for apartment classification. Thus, correlation of outdoor and indoor temperature could be strongest with a time delay of a few hours in one apartment but with a time delay of several hours in another, depending on the thermal mass or overall thermal transmission of the building. The distributions of TMC were assessed between buildings and apartment types.

### Definition of unhealthy home temperature

In Finland, health risks of the home environment are regulated by the Ministry of Social Affairs and Health, according to which acceptable home temperature during the heating season is between + 18 °C and + 26 °C (Finnish Ministry of Social Affairs and Health 2015). The latest Cold Weather Plan for England recommends keeping home temperatures above + 18 °C in winter (Katiyo et al. 2018). Standard EN 16798-1:2018 (formerly EN 15251:2007) gives limit values in four categories: (i) 21 °C to 25 °C; (ii) 20 °C to 25 °C; (iii) 18 °C to 25 °C; and (iv) 17 °C to 25 °C (European Committee for Standardization European Committee for Standardization 2018). World Health Organization (WHO) has recommended a minimum indoor temperature of + 18 °C and 2–3 °C warmer for rooms occupied by elderly persons (WHO 1987).

In order to assess how apartments in the different categories performed in terms of healthy living during cold weather, we adopted a priori for our analyses the lower thresholds of + 18 °C and + 20 °C, and higher threshold of + 25 °C, combining the abovementioned standards while taking into account the Finnish building stock. We evaluated the occurrence, frequency, and duration of episodes with temperatures above/below the thresholds. Degree hours above/below threshold were calculated. For example, indoor temperature of + 17 °C for 2 h (1°× 2 h) would result in the same 2 Kh as indoor temperature of + 16 °C for 1 h (2 K × 1 h). If both episodes would occur in the same apartment, the apartment-specific total degree hours would be 4 Kh (2Kh + 2Kh). Although degree hours may not accurately describe health effect-inducing exposures of residents, the concept is widely used in building physics and occupational health research and adopted in European standards (European Committee for Standardization 2008).

### Analyses on the influence of orientation and floor

We investigated whether compass point orientation or floor of the apartment influenced its apartment type, i.e., whether some types were more predominant in some orientations or floors. This could be the case due to exposure to solar radiation or predominant wind, for example. Orientation was simply defined as the compass point of the one façade or, in case of two perpendicular external walls, as the compass point of the corner, i.e., inverse vector of two equally sized complementary 45° angles between the perpendicular walls. For example, an apartment with one outer wall toward northeast and one outer wall toward northwest was defined as having main orientation toward north. Apartments with incomparable or complex orientations (49 apartments out of 417), such as main façades facing both north and south or 3 or more directions, were excluded from this analysis. Apartments with main orientation toward southeast, south, southwest, or west were classified as being exposed to direct sunlight. Apartments facing northwest, north, northeast, or east were classified as not being exposed to direct sunlight. We also compared first, top, and other floors within and between apartment types. All analyses were conducted using R version 3.3.2, and SPSS version 26.0.0.0.

## Results

Outdoor temperatures during the study period ranged from − 20.4 °C to + 14.0 °C, with median − 3.35 °C and mean − 3.87 °C (SD 5.47). There were no relevant differences in the outdoor temperature distributions between apartment types (data not shown). The heating degree days in the city of Kotka was 3906 (base 17 °C) for the year 2018 and 1787 for Feb–Apr 2018.

The residential buildings in the study were constructed between years 1955 and 2008 and included 12 to 53 apartments each in 2 to 8 floors. The construction material was either concrete (10 buildings) or brick (4 buildings). Mechanical exhaust ventilation was more common than natural ventilation. Table 1 shows characteristics of the buildings in the study.

**Table 1:** Characteristics of the residential buildings in the study

Building ID	Construction year	Floors (*n*)	Floor area (m^2^)	Building material	Ventilation type	Energy consumption (kWh/m^3^/year)
1	1969	3	1356	Concrete	Mechanical^a^	44.0
2	1955	7	2750	Brick	Mechanical	48.1
3	1982	3	3587	Concrete	Mechanical	39.3
4	1997	6	2094	Concrete	Mechanical	37.2
5	1964	6	2548	Concrete	Natural^b^	45.2
6	1974	3	1000	Brick	Natural	54.3
7	1974	5	3270	Brick	Natural	36.2
8	1974	3	1000	Brick	Mechanical	54.3
9	1988	5	1478	Concrete	Mechanical	48.0
10	1962	3	1023	Concrete	Mechanical	45.5
11	2008	8	3819	Concrete	Mechanical	23.2
12	1983	2	2789	Concrete	Natural	50.9
13	1983	2	1200	Concrete	Mechanical	44.7
14	1985	2	1200	Concrete	Mechanical	44.7

Energy consumption is that of the year 2016

^a^Mechanical, mechanical exhaust ventilation. ^b^Natural, natural ventilation

There were no statistically significant differences in mean indoor temperatures between buildings (+ 21.9 °C to + 23.2 °C). The building-specific average indoor temperature range varied from 1.73 °C (SD 0.49) to 3.89 °C (SD 3.25). Table 2 summarizes the indoor temperature parameters of the buildings in the study.

**Table 2:** Distributions of the indoor temperature parameters and apartment types in the buildings

Building ID	T mean (SD)	T range (SD)	CCC^a^	TMC^b^ range (h)	TMC^b^ quartiles (h)	Under-controlled apartments, *n* (%)	Controlled apartments, *n* (%)	Over-controlled apartments, *n* (%)
1	22.16 (0.87)	2.71 (1.72)	0.07	0.0–95.5	11.0, 30.0, 50.5	7 (38.9)	5 (27.8)	6 (33.3)
2	21.93 (0.93)	3.13 (1.27)	0.25	0.0–96.0	12.0, 39.5, 55.5	34 (64.2)	8 (15.1)	11 (20.8)
3	22.45 (0.83)	1.73 (0.49)	− 0.11	0.0–97.5	10.5, 13.0, 38.0	2 (4.7)	34 (79.1)	7 (16.3)
4	22.64 (0.91)	3.24 (1.17)	− 0.29	0.0–57.0	0.5, 5.0, 10.5	9 (25.7)	4 (11.4)	22 (62.9)
5	22.53 (0.79)	2.75 (0.90)	0.22	0.0–90.5	9.5, 21.0, 66.5	15 (53.6)	7 (25.0)	6 (21.4)
6	22.91 (0.59)	2.78 (0.72)	0.20	0.0–99.5	5.0, 9.5, 28.5	20 (54.1)	5 (13.5)	12 (32.4)
7	22.40 (0.66)	2.90 (0.79)	0.42	0.0–47.0	1.0, 9.5, 23.0	19 (86.4)	2 (9.1)	14 (63.6)
8	23.15 (1.08)	2.90 (0.66)	0.44	0.0–44.0	10.0, 23.0, 31.0	11 (91.7)	1 (8.3)	0 (0.0)
9	22.71 (0.76)	2.65 (1.19)	0.05	0.0–85.5	9.0, 21.5, 39.0	12 (29.3)	15 (36.6)	0 (0.0)
10	22.12 (1.04)	3.89 (3.25)	− 0.48	0.0–88.0	1.0, 3.3, 5.9	1 (6.3)	2 (12.5)	13 (81.3)
11	22.39 (0.63)	2.28 (0.62)	− 0.12	0.0–93.5	5.5, 11.0, 43.0	8 (24.2)	16 (48.5)	9 (27.3)
12	22.10 (1.32)	2.78 (0.71)	0.12	0.0–91.0	8.0, 28.0, 51.0	16 (50.0)	4 (12.5)	12 (37.5)
13	21.95 (1.08)	2.40 (1.22)	0.10	0.0–99.5	9.5, 23.0, 66.5	5 (20.8)	13 (54.2)	6 (25.0)
14	22.58 (0.98)	2.69 (1.38)	0.04	0.0–99.5	1.1, 16.8, 58.9	5 (20.8)	10 (41.7)	9 (37.5)

Distributions of apartment types, indoor temperatures, and building-specific correlation parameters between outdoor and indoor temperatures of the residential buildings in the study. Mean temperature is the mean of all temperature recordings in all apartments of the building

^a^CCC, cross-correlation coefficient. ^b^TMC, time to maximum correlation between outdoor temperature and indoor temperature, in hours

Table 2 shows the prevalence of the different apartment types in the buildings. Twelve of the 14 buildings included apartments of all three types, while 2 buildings included only type 1 and type 2 apartments. There was heterogeneity in the building-level CCC:s, which reflected the distributions of apartment types in the buildings (i.e., CCC 0.44 in a building with 91.7% of the apartments being type 1, and CCC − 0.48 in a building with 81.3% of the apartments being type 3). There were pronounced differences in TMC between buildings.

Table 3 shows the indoor temperature characteristics of the apartments by the three apartment types. There were no practical differences in the mean temperatures (+ 22.41 °C, SD 0.94) between types. The indoor temperature range was, by definition, narrow in controlled apartments (median 1.78 °C, SD 1.29) and wider in under-controlled and over-controlled apartments (3.03 °C, SD 0.99, and 3.25 °C, SD 1.04, respectively). There was substantial heterogeneity in the median TMC, ranging from 0 to 99.5 h.

**Table 3:** Distributions of the indoor temperature parameters by apartment type

Parameter	Under-controlled apartments	Controlled apartments	Over-controlled apartments	All apartments
Mean temperature (SD)	22.29 (0.99)	22.33 (0.87)	22.65 (0.90)	22.41 (0.94)
Temperature range (SD)	3.03 (0.99)	1.78 (1.29)	3.25 (1.04)	2.72 (1.26)
CCC^a^ mean (SD)	0.48 (0.12)	0.11 (0.52)	− 0.53 (0.19)	0.06 (0.53)
TMC^b^ (h)				
Minimum	0.0	0.0	0.0	0.0
1st quartile	5.0	9.0	2.0	5.0
Median	24.5	19.5	9.5	13.0
Mean	32.5	27.5	17.5	26.5
3rd quartile	51.5	46.0	13.0	45.0
Maximum	97.5	99.5	99.5	99.5

Distributions of indoor temperatures, correlation parameters between outdoor and indoor temperatures, and time delays of correlation, by apartment type ^a^CCC, cross-correlation coefficient. ^b^TMC, time to maximum correlation between outdoor temperature and indoor temperature, in hours

**Fig. 1:**
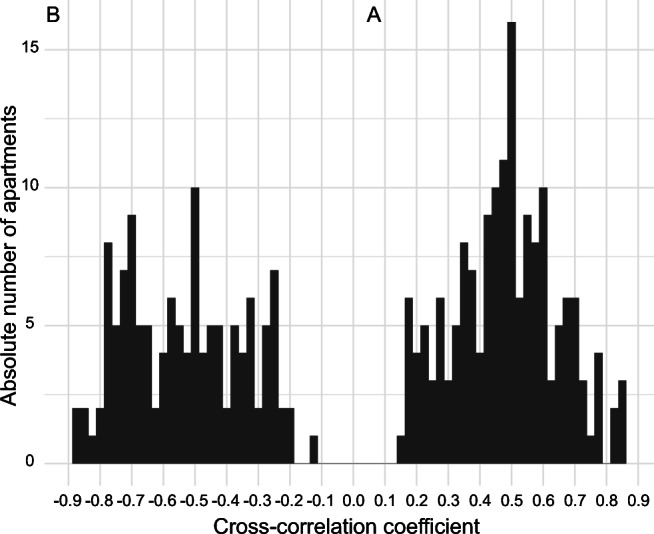
Absolute number of apartments by apartment-specific cross-correlation coefficients. The two peaks illustrate two opposite apartment types, over-controlled on the left with negative correlations, and under-controlled on the right with positive correlations between outdoor and indoor temperatures. Controlled apartments have not been omitted from the histogram, which displays the cross-correlation coefficients of all apartments in the study

The absolute number of apartments by CCC value is presented in Fig. 1, where the distributions of under-controlled and over-controlled apartments are represented by the positive and negative correlations between outdoor and indoor temperatures.

**Fig. 2:**
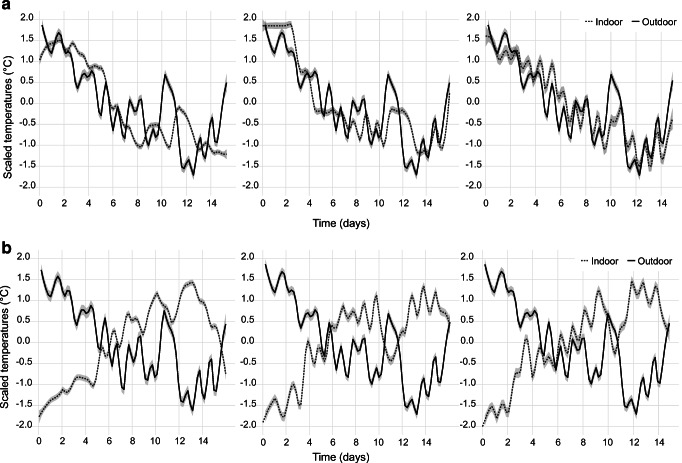
**A.** Time-series of outdoor and indoor temperatures in select under-controlled apartments**, B.** Time-series of outdoor and indoor temperatures in select over-controlled apartments**.** The Y-axis has been normalized to improve readability

Figure 2 shows normalized time-series of outdoor and indoor temperatures in select individual apartments. The 3 apartments in panel A are under-controlled: when the outdoor temperature decreases, indoor temperature decreases (and vice versa). The 3 apartments in panel B are over-controlled: when the outdoor temperature decreases, indoor temperature increases (and vice versa).

Episodes with indoor temperature below + 18 °C were rare in our data and occurred in 4.3%, 0.8%, and 5.5% of the under-controlled, controlled, and over-controlled apartments, respectively. Maximum 18.4% of total time (208 h) was manifested with temperatures below +18 °C in one under-controlled apartment (Supplementary Table 1). Indoor temperatures below + 20 °C occurred in 26.2%, 7.9%, and 23.6% of the apartments in under-controlled, controlled, and over-controlled apartments, respectively. While these episodes most commonly lasted between 2 to 6 h, there were longer episodes up to 258 h (under-controlled), 44 h (controlled), and 73 h (over-controlled apartments). In one controlled apartment, 69% of the total time was manifested with temperatures below the threshold. For under-controlled and over-controlled apartments, a maximum of 54% and 38% of total time was manifested with temperatures below the + 20 °C threshold (Supplementary Table 2).

A handful of apartments were manifested with episodes of indoor temperatures above + 25 °C (Supplementary Table 3). A maximum of 13 such episodes, with median duration of 1.6 h (Q1 0.5, Q3 2.1), occurred in under-controlled apartments. A maximum of 8 heat episodes with median duration of 2.8 h (Q1 1.4, Q3 5.3) occurred in over-controlled apartments. There were no heat episodes in controlled apartments. The longest time with indoor temperature exceeding + 25 °C is 78 h or 5.9% of total time (over-controlled apartment, Supplementary Table 3).

Distributions of main compass point orientation and floor by apartment type are presented in Supplementary Table 4. There were no statistically significant differences in the distributions of these factors between the under-controlled, controlled, and over-controlled apartments.

## Discussion

This proof-of-concept study demonstrates that not all apartments react similarly to changes in outdoor temperature. We implemented a straightforward apartment typing based on the correlation between outdoor and indoor temperatures. Under-controlled apartments cooled down when outdoor temperature decreased. Over-controlled apartments warmed up as outdoor temperature decreased. Controlled apartments remained within a narrow temperature range regardless of changes in outdoor temperature. Thermal exposures outside the recommended limits occurred in all apartment types. Episodes of overheating occurred but were rare. Floor and orientation of an apartment did not explain its type. Different types of apartments were present in all buildings, which implies that it may be difficult to use building-specific characteristics to predict apartment-specific thermal conditions.

### Strengths and limitations

A major strength of this study was demonstrating conceptual differences between apartment types in their reaction to cold weather. Although on average less than 8% of time is spent outdoors (Klepeis et al. 2001; Brasche and Bischof 2005; Matz et al. 2015; Mäkinen et al. 2006), weather continues to be a strong evidence-based predictor of population-level health effects (Toloo et al. 2013; Vicedo-Cabrera et al. 2018). It is not known what the most harmful places or patterns of exposures during the harmful weather events are (Ryti et al. 2016). Meteorological alarm systems are founded on shared measurements of outdoor temperatures, and the premise of an alarm system is that it leads to some sort of protective action when triggered. It is important to consider whether advice for such protective action can be applied to the entire population or whether following it may promote paradoxical exposures in some instances. We demonstrated with a simple analysis that this topic may need to be revisited.

We included a relatively large number of randomly selected apartments in the analyses from fourteen apartment buildings. Heat distribution systems were similar in all apartments. The measurements were designed to have minimal impact on the lives of the residents to reduce any bias from intervention. We focused on evaluating changes in temperature, taking advantage of the high time resolution in the data and the intercomparability of the time-series in each apartment. The temperature measurements were conducted following a standardized protocol and identical and calibrated equipment.

There are several limitations of the study. We did not perform complex modeling of the human microenvironment, and neither did we conduct engineering analysis of the buildings or heating systems in the study. Instead, we looked at one potential health determinant (indoor temperature) and its association with a known predictor of population-level health effects (outdoor temperature). However, this was sufficient to answer our study question. Second, we were able to gather data on two winter months only. For this reason, we cannot make inference on the correlations of outdoor and indoor temperatures during other seasons or possible trends in the correlations. It is not possible to conclude that apartments manifesting as under-controlled in our data would not manifest as over-controlled or controlled during other seasons. Some of our choices in apartment classification can be criticized. Selecting a 2 °C indoor temperature range as relevant for health effects may be arbitrary, but there is no clear evidence for a more relevant range.

We allowed the time to maximum correlation vary between apartments. While this has several strengths, it also means that when looking at different time spans, a positive CCC might, at least theoretically, become negative. In fact, we find it likely that most apartments immediately cool to some extent when outdoor temperature declines, and then, some continue cooling, adjust back to normal, or overheat as a response. Clarifying the different time spans would be an interesting topic for future studies, but it needs a thorough engineering analysis of the buildings and heating systems. Finally, a limitation of the study is that we could not randomly sample apartments for the study to represent Finnish building stock. However, no apparent biases were discovered in scrutiny of the recruiting process. Even though the results cannot be safely generalized over time or space, they are important in a conceptual level and prove that major differences in apartment reactions to cold weather exist.

### Interpretation of the results

Several factors could explain the observed heterogeneity in the way apartments react to cold weather. The underlying reason for room temperature variation in general is an imbalance between heat supplied to a room and heat losses of the room. Heating needs of the rooms do not increase or decrease in the same proportion with outdoor temperature. This is due to differences in time constants of the rooms (i.e., relation between thermal capacity and thermal conductance). Area of external walls, windows, outdoor air leakage, and inadequate thermal insulation will decrease the time constant, making rooms cool more rapidly (coupled with the need to heat more rapidly) than rooms with high time constant and less external walls, less windows, and less outdoor air leakage. Compass point orientation and floor number may also influence the indoor thermal microclimate, as these are related to the amount of solar radiation and exposure to wind. Such a phenomenon has previously been reported during the summer (Langner et al. 2014), although building thermodynamics are different during the heating season and summer. At any rate, these factors did not explain the heterogeneity or the apartment groupings in our study. We believe that this illustrates the complexity of the topic and that more evidence is needed before shared outdoor temperatures can be used as predictors of indoor temperatures in individual apartments.

Differences between construction materials probably explain little of the observed heterogeneity. Thermal capacity does not vary substantially between different masonry materials, and brick is usually reserved for the exterior caver of external walls, which is outside the thermal insulation.

Supply water to the radiator network is usually controlled by outdoor temperature according to building characteristics. Temperature levels are often set to minimize the number of apartments that are too cold, which may lead to a greater number of over-controlled apartments in the building. Control systems for heating also often include a parameter for wind velocity to compensate for the effect of air leakage on the heating need. If the wind parameter is not included in the control algorithm, apartments in the wind side of the building may manifest as under controlled.

The balancing of the hot water distribution system in the design conditions is a demanding and time-consuming operation. We did not have an opportunity to check the quality of the balancing work. Some of the buildings are quite old, and there may be some refurbishments during the life time of the buildings, like new windows, better air tightness of windows, extra thermal insulation, or wet thermal insulations. The heating network should have been balanced frequently. Although our data included the year of the latest heating system adjustment for most buildings (data not shown), we did not have details on the actual balancing or the changes in the thermal insulation of rooms.

Radiators were the sole means of heat emission in all apartments. According to common practice in Scandinavia, the number and power of radiators are calculated during the planning phase of the buildings, taking into account the thermal characteristics of each room. The design water flow supplied to the radiators has supposedly been balanced at the end of the construction phase, and it is based on apartment-specific measurements during cold weather conditions (guideline value of − 5 °C or below), which is also a common practice in Scandinavia. The target value of indoor room temperatures was + 21 °C before the occupants moved in, with the radiator thermostats inactive or not attached yet. Radiator thermostats were then installed, and their main role is to limit overheating of a room when indoor temperature of the thermostat reaches threshold level set by the resident. However, the heating system reacted only to the set point values lower than + 21 °C, not higher.

Several aspects of this type of heat emission may generate differences in how different apartments react to cooling weather. The thermostat radiator valve is an inexpensive device with varying quality between the brands and even within brands. The performance of the valves may be one reason for variation of room temperature control. For example, the operation of the valve may be affected by the supply water temperature in the radiator circuit due to heat conduction between the valve and thermostat. Also, the position of the thermostat influences the operation of the device: if the thermostat is installed in vertical position on the valve, it is affected by convection from the valve. In horizontal position, the valve is more likely to sense the actual room air temperature, but furniture, curtains, and cold air currents from open windows may still cause inadequate operation of the thermostat. In this study, we did not have a possibility to investigate these factors, which should be topics of the future studies.

One explanation for the observed differences between apartments may also be related to altered performance of radiators over time. Water circulation systems of radiators are prone to clogging, which decreases the amount of thermal energy released in a unit of time at the radiator-air interface. This could lead to lowered baseline temperature in a room or weakened responsiveness to cooling air sensed by the thermostat. Such a phenomena might be present in under-controlled apartments. Another consequence of a clogged radiator is that, at least theoretically, the water flow in other radiators in the same network is increased due to the increased resistance of the clogged radiator. This may overheat other rooms in the same circuit, leading to increased temperature differences between rooms, which could partly explain associations in over-controlled apartments. According to the same principle, clogging of one radiator could also influence the heating in other apartments of the same building, depending on how the water circulation is arranged. Radiator networks used to be designed so that radiators in a room are connected to the same risers, not with other radiators in another apartments of the same floor. In such a setting, it is particularly interesting to consider whether a situation with high wind velocity and clogging of some radiators in the wind side of the building could influence the temperature reactivity of the other apartments in wind side, and to what extent.

Some thermostats may have been mishandled by the occupants over the years. In general, human behavior is likely to substantially influence the indoor thermal conditions during weather events. Some residents may keep their windows open amidst of winter for purposes of letting in outdoor air or regulating uncomfortably hot indoor temperatures, which would further stimulate the thermostats (Keatinge 1986). Thermostats may also be covered with curtains or furniture, leading to a situation where the temperature of the thermostat differs greatly from the room temperature. This might contribute to an under-controlled performance of the apartment. Changes and differences in relative humidity and thermal comfort may also influence human behavior. Although we did not have any data on behavior of the residents, irregularities and outliers in the otherwise consistent data were present and suggested human action. However, if such action was abundant, the CCC:s should be closer to 0 than 1 or – 1, and there should be major variations in indoor temperature, but the number of apartments not correlating with the outdoor conditions is low (Fig. 1).

Although it is difficult to ascertain which of the abovementioned reasons are most important in explaining why apartments may react differently to cooling outdoor temperatures, they all justify our hypothesis and support the case of critical evaluation of generalized public health advice during weather events.

### Implications

Although ours is a single and relatively short study, empirical demonstration of the conceptual differences between apartments raises important public health questions. While substantial efforts are being made to reduce winter mortality related to inadequate housing, heating, and fuel poverty (Marmot Review Team 2011; Katiyo et al. 2018), it is generally assumed that there is a positive correlation between outdoor and indoor temperatures. Potential health effects due to overheating during the heating season have not been widely recognized as an issue in scientific literature. The variation in time delay between the change in outdoor temperature and the change in indoor temperature in different apartments also means that synchronized measures of adjusting the heating could amplify the temperature differences between baseline and end result, leading to harmful low or high home temperatures in more apartments. Most importantly, shared weather parameters continue to play the most important role in forecasting public health problems related to thermal exposure (Toloo et al. 2013; Pachauri and Meyer 2015). Our study questions whether individual exposure indoors can be predicted from these shared weather parameters. If this is not the case, exposures during over 90% of the average potential exposure time remain unpredictable (Klepeis et al. 2001; Brasche and Bischof 2005; Matz et al. 2015). One may ask that if we do not know the actual exposure profiles during the weather events, how well do we really understand weather-related pathogenesis, and how effective can we be at stopping it?

## Conclusions

This proof-of-concept study demonstrated that while some apartments may cool during cold weather, some apartments may overheat during the same events. This concept is important for public health professionals and scientists working with weather-related mitigation and adaptation programs. In particular, the concepts presented in this paper have major theoretical implications for the construction of meteorological alarm systems that are founded on shared exposure data.

Furthermore, we demonstrated that there is an urgent need for improvement of temperature control of heating systems of apartment buildings, as too warm and too cold apartments are likely to induce adverse health effects. The results show that the temperature control does not work as intended, and building industry should develop and build better systems for automatic temperature control of buildings which could also use weather forecasts to improve the temperature control. Over-controlled apartments waste heating energy, and need for improvements in the control of heating systems was also recognized in the recently revised Energy Performance of Buildings Directive (Directive (EU) 2018/844 of the European Parliament), which requests for better control of heating in all EU members states. Building owners should regularly check the temperature control of the heating systems.

It would be desirable to link forecasts of harmful weather with advice on how to behave at home to avoid adverse health effects. Our study indicates that further research is needed for this to happen effectively and safely. More elaborate conceptualizations of everyday thermal exposures are needed to fully understand how to reduce risks associated with the interplay between thermal microenvironment and the human body.

## Electronic supplementary material

ESM 1 (DOCX 33 kb)
