# Supplementary material for: Paradoxical home temperatures during cold weather: a proof-of-concept study
Source: Int J Biometeorol. 2020 Aug 27;64(12):2065–76. doi: 10.1007/s00484-020-01998-7 (PMC7658083; doi:10.1007/s00484-020-01998-7)
Supplement: Supplementary file 1 — (DOCX 33 kb) [file 484_2020_1998_MOESM1_ESM.docx]

**SUPPLEMENTARY INFORMATION**

**Supplementary Table 1. Episodes of indoor temperature below +18°C.**

| **Parameter** | **Under-Controlled Apartments** | **Controlled Apartments** | **Over-Controlled Apartments** |
| --- | --- | --- | --- |
| Number of Apartments with Episodes, n (%) | 7 (4.3) | 1 (0.8) | 7 (5.5) |
| Number of Episodes per Apartment |  |  |  |
| Min, Max | 1, 5 | 1, 1 | 1, 2 |
| Median (Q1, Q3) | 1 (1, 3) | 1 (1, 1) | 1 (1, 1) |
| Duration of Episodes per Apartment (h) |  |  |  |
| Min, Max | 0, 42 | 26.5, 26.5 | 0.5, 1.5 |
| Median (Q1, Q3) | 1 (0, 8) | 26.5, (26.5, 26.5) | 0.5 (0.5, 0.5) |
| Total time exceeding threshold per Apartment, h (%) |  |  |  |
| Min | 0 (0) | 26.5 (1.8) | 0.5 (0.04) |
| Max | 208 (18.4) | 26.5 (1.8) | 1.5 (0.12) |
| Median | 1 (0.1) | 26.5 (1.8) | 0.5 (0.04) |
| Q1, Q3 | 0, 21 (0.0, 1.3) | 26.5, 26.5 (1.8, 1.8) | 0.5, 1.5 (0.04, 0.06) |
| Degree Hours per Apartment |  |  |  |
| Min, Max | 0, 108243 | 20866, 20866 | 0.3, 8.8 |
| Median (Q1, Q3) | 4 (0, 1352) | 20866 (20866, 20866) | 0.4 (0.4, 1.5) |

Degree Hours were calculated multiplying the time (in hours) below threshold and degrees (in Kelvin) below threshold.

**Supplementary Table 2. Episodes of indoor temperature below +20°C.**

| **Parameter** | **Under-Controlled Apartments** | **Controlled Apartments** | **Over-Controlled Apartments** |
| --- | --- | --- | --- |
| Number of Apartments with Episodes, n (%) | 43 (26.2) | 10 (7.9) | 30 (23.6) |
| Number of Episodes per Apartment |  |  |  |
| Min, Max | 1, 49 | 1, 101 | 1, 33 |
| Median (Q1, Q3) | 6 (2, 15) | 16 (2, 41) | 3 (1, 6) |
| Duration of Episodes per Apartment (h) |  |  |  |
| Min, Max | 0, 258 | 0, 44 | 0, 73 |
| Median (Q1, Q3) | 6 (2, 14) | 3 (1, 6) | 2 (1, 4) |
| Total time exceeding threshold per Apartment, h (%) |  |  |  |
| Min | 0 (0) | 0 (0) | 0 (0) |
| Max | 572 (54) | 1021 (69) | 600 (38) |
| Median | 43 (4) | 68 (6) | 6 (0.04) |
| Q1, Q3 | 4, 195 (0.0, 16) | 2, 447 (0.0, 40) | 2, 13 (0.04, 1) |
| Degree Hours per Apartment |  |  |  |
| Min, Max | 0, 385305 | 0, 2693286 | 0, 564300 |
| Median (Q1, Q3) | 884 (21, 42173) | 2021 (6, 111327) | 30 (7, 106) |

Degree Hours were calculated multiplying the time (in hours) below threshold and degrees (in Kelvin) below threshold.

**Supplementary Table 3. Episodes of indoor temperature above +25°C.**

| **Parameter** | **Under-Controlled Apartments** | **Controlled Apartments** | **Over-Controlled Apartments** |
| --- | --- | --- | --- |
| Number of Apartments with Episodes, n (%) | 5 (3.1) | 0 (0) | 5 (3.9) |
| Number of Episodes per Apartment |  |  |  |
| Min, Max | 1, 13 | 0, 0 | 3, 8 |
| Median (Q1, Q3) | 1 (1, 7) | 0 (0, 0) | 5 (4, 6) |
| Duration of Episodes per Apartment (h) |  |  |  |
| Min, Max | 0.5, 3 | 0, 0 | 1.2, 19.5 |
| Median (Q1, Q3) | 1.6 (0.5, 2.1) | 0 (0, 0) | 2.8 (1.4, 5.3) |
| Total time exceeding threshold per Apartment, h (%) |  |  |  |
| Min | 0.5 (0.03) | 0 (0) | 4 (0.3) |
| Max | 20.5 (1.78) | 0 (0) | 78 (5.9) |
| Median | 3 (0.23) | 0 (0) | 16 (1.4) |
| Q1, Q3 | 0.5, 15 (0.05, 1.12) | 0, 0 (0, 0) | 11, 26 (1.0, 2.2) |
| Degree Hours per Apartment |  |  |  |
| Min, Max | 14, 3494 | 0, 0 | 65, 4243 |
| Median (Q1, Q3) | 709 (172, 902) | 0 (0, 0) | 232 (175, 747) |

Degree Hours were calculated multiplying the time (in hours) above threshold and degrees (in Kelvin) above threshold.

**Supplementary Table 4. Distributions of main compass point orientation and floor of apartments by apartment type.**

| **Parameter** | **Under-Controlled Apartments, n(%)** | **Controlled Apartments, n(%)** | **Over-Controlled Apartments, n(%)** | **Chi-square** |
| --- | --- | --- | --- | --- |
| Main compass point orientation |  |  |  | 0.323 |
| North | 24 (16.2) | 12 (10.8) | 14 (12.8) |  |
| Northeast | 11 (7.4) | 8 (7.2) | 9 (8.3) |  |
| East | 19 (12.8) | 22 (19.8) | 14 (12.8) |  |
| Southeast | 11 (7.4) | 9 (8.1) | 11 (10.1) |  |
| South | 16 (10.8) | 22 (19.8) | 21 (19.3) |  |
| Southwest | 32 (21.6) | 15 (13.5) | 23 (21.1) |  |
| West | 16 (10.8) | 14 (12.6) | 6 (5.5) |  |
| Northwest | 19 (12.8) | 9 (8.1) | 11 (10.1) |  |
| Exposure to direct sunlight |  |  |  | 0.689 |
| Yes | 75 (50.7) | 60 (54.1) | 61 (56.0) |  |
| No | 73 (49.3) | 51 (45.9) | 48 (44.0) |  |
| Floor |  |  |  | 0.057 |
| First | 33 (22.3) | 30 (27.0) | 30 (27.5) |  |
| Other | 90 (60.8) | 50 (45.0) | 62 (56.9) |  |
| Top | 25 (16.9) | 31 (27.9) | 17 (15.6) |  |
| Total | 148 (100.0) | 111 (100.0) | 109 (100.0) |  |

Main compass point orientation was defined as the compass point of the one façade or, in case of two perpendicular external walls, as the compass point of the corner. Apartments with incomparable or complex orientations (49 apartments out of 417) were excluded from this analysis. Exposure to direct sunlight was defined as main orientation towards southeast, south, southwest or west. Chi-square tests were significant at 0.05.
